# Genetic analysis of efficiency-related traits in Boer x Central Highland goats

**DOI:** 10.1371/journal.pone.0305749

**Published:** 2024-07-10

**Authors:** Zeleke Tesema, Kefyalew Alemayehu, Tesfaye Getachew, Damitie Kebede, Mekonnen Tilahun, Belay Deribe, Mesfin Lakew, Getachew Worku Alebachew, Mengistie Taye, Solomon Gizaw

**Affiliations:** 1 Debre Birhan Agricultural Research Center, Debre Birhan, Ethiopia; 2 College of Agriculture and Environmental Sciences, Bahir Dar University, Bahir Dar, Ethiopia; 3 International Center for Agricultural Research in the Dry Areas (ICARDA), Addis Ababa, Ethiopia; 4 Andasa Livestock Research Center, Bahir Dar, Ethiopia; 5 Sirinka Agricultural Research Center, Woldia, Ethiopia; 6 Amhara Agricultural Research Institute, Bahir Dar, Ethiopia; 7 International Livestock Research Institute (ILRI), Addis Ababa, Ethiopia; INRAE, FRANCE

## Abstract

This study aimed to identify important non-genetic factors and estimate genetic parameters for efficiency-related traits in Boer x Central Highland goats. The genetic parameters were estimated using the Average Information Restricted Maximum Likelihood algorithm using the WOMBAT program fitting animal model. The least-squares means for growth efficiency from birth to 3 months (GE1), 3–6 months (GE2), 6–12 months (GE3), relative growth rate from birth to 3 months (RGR1), 3–6 months (RGR2) and 6–12 month (RGR3) were 294.0 ± 5.06, 36.6 ± 1.20, 44.9 ± 1.81, 1.46 ± 0.01, 0.32 ± 0.01 and 0.19 ± 0.01, respectively. Birth type, blood level, sex of the kid, and year of kidding had a sizable effect on efficiency-related traits. About 18, 3.0, 23, 20, and 12% of the phenotypic variation in GE2, GE3, RGR1, RGR2, and RGR3 was explained by the direct additive genetic effect. Except for RGR3, all investigated traits were under the influence of maternal genetic effect, and maternal heritability ranged from 0.09 to 0.17. The total heritability estimate depicts that slow genetic progress would be expected from selection. Nevertheless, even with this level of heritability, selection for efficiency-related traits would improve the efficiency of chevon production as these traits are economically important traits. Nearly six-months of age was when farmers sold Boer crossbred goats. Therefore, improving the growth efficiency till the marketing age (GE2) in such a scenario could increase the production efficiency.

## Introduction

Genetic improvement of indigenous Central Highland goats through crossing with the productive Boer goat has been conducted since 2007. The crossbreeding program aimed to improve the growth performance and meat production and thereby improve the livelihood of farmers. Accordingly, crossbred bucks with 50% exotic blood level were disseminated to farmers and crossed with indigenous Central Highland goats. Crossbred goats need better management for survival and productivity [1]. In low-input systems, however, producers aim to optimize production using the least possible production inputs to achieve the highest possible output rather than using possible levels of inputs to attain the highest possible output [2]. Thus, the productivity of goats is highly dependent on their efficiency to use the available resources to which they are subjected. In addition, farmers sold crossbred goats near six months of age. In such a situation, improving the growth rate up to the marketing age could enhance the production efficiency and farmers’ economy.

Selection for growth traits is the option to enhance productivity of goats. But sometimes, selection for better body weight could be associated with high mature body weight if animals are maintained for breeding, which results in high cost. However, selection for relative growth rate and growth efficiency increases early growth independent of the mature size and is suggested as an approach to enhance production efficiency and reduce the production cost of animals [3–5]. Besides, efficiency-related traits such as growth efficiency and relative growth rate are positively correlated with feed efficiency traits according to previous studies [6–13]. In order to breed for feed efficiency, records of feed intake and production must also be kept. But the recording of feed intake of individual animal is not obvious, particularly in developing countries. Therefore, improvements in growth performance and feed efficiency could be made through the selection of animals for efficiency-related traits.

Identification of the possible non-genetic factors and estimation of the genetic parameters (variance, heritability, and genetic correlations) for efficiency-related traits are the pre-requisite for genetic improvement through selective breeding in conjunction with crossbreeding. Although there are several studies on growth efficiency and relative growth rate of cattle [5, 6, 8–10] and sheep [11, 12, 14], these traits have not been studied so far in goats except for Mokhtari et al. [13]. Therefore, the objectives of this study were to identify non-genetic factors and to estimate genetic parameters for efficiency-related traits in Boer x Central Highland goats.

## Material and methods

### Data and flock management

Data were collected from an experimental breeding flock of Boer x Central Highland goats, maintained at Sirinka Agricultural Research Center small ruminant breeding, evaluation, and dissemination site, which is located at an altitude of 1850 m.a.s.l and 11°45’ 00" N and 39°36’ 36" E. The mean annual rainfall is 950 mm. The area is a moderately warm temperature zone with mean daily temperature ranges of 16–21°C. Data were collected from 2009 to 2018. A detailed description of the data is presented in Table 1. The animals were reared under semi-intensive management i.e., animals were allowed to graze for six hours per day and sheltered in a semi-open concert barn during the night based on their physiology, sex, and age. When returned from gazing/browsing, flocks were supplemented with a concentrate mix (100–400 g day^-1^ based on their physiology and age) comprised of Wheat bran, Noug cake, and salt as a mineral source. Goats were vaccinated for common diseases in the area and treated regularly. All experimental techniques and animal care were in line with FASS [15], and animal nutrition and health researchers of Sirinka Agricultural Research Center, Amhara Region Agricultural Research Institute, confirmed this during annual review forum.

**Table 1:** Description of data structure for efficiency-related traits in Boer crossbred goats.

Items	Traits					
	GE1	GE2	GE3	RGR1	RGR2	RGR3
Number of records	674	533	350	674	533	350
Number of sire	22	21	20	22	21	20
Number of dam	219	191	163	219	191	163
Number of progeny/ sire	30.6	25.4	17.5	30.6	25.4	17.5
Number of progeny/ dam	3.07	2.79	2.15	3.07	2.79	2.15
Mean	293.5	36.6	46.9	1.46	0.32	0.19
SD	131.4	27.7	33.5	0.36	0.21	0.11
CV %	39.0	22.0	27.2	37.1	28.8	38.8

GE1, growth efficiency from birth to weaning; GE2, growth efficiency from weaning to six months; GE3, growth efficiency from six- month to yearling age; RGR1, relative growth rate from birth to weaning; RGR2, relative growth rate from weaning to six months; RGR3, relative growth rate from six- month to yearling age

The mating method was a natural controlled mating method. One buck was assigned to 20–30 breeding females, and the bucks were kept with does for forty-five days. During mating, herdsmen were assigned to each mating group to collect the mating data and pedigree information. The pure Central Highland does were crossed with pure Boer bucks to produce the F1 crossbreds with 50% Boer blood level. Male and female crossbreds were *inter se mated* to produce F2 crossbreds. About 25% of female crossbreds with 50% blood level were mated with 75% blood level and pure Boer buck to increase their exotic blood level to 62.5% and 75%, respectively. As a limitation, the sample size and data structure may influence the interpretation of the findings from this study to some extent. Indeed, the data was collected by researchers under on-station management of animals to improve the data quality, and this may reduce the impact of data size on genetic parameter estimates.

### Evaluated traits

The investigated traits include growth efficiency from birth to weaning (GE1) = ((weaning weight–birth weight)/birth weight) x 100, from weaning to six months (GE2) = ((six-month weight–weaning weight)/weaning weight) x 100 and growth efficiency from six-month to yearling age (GE3) = ((yearling weight–six-month weight)/six-month weight) x 100. Besides, the relative growth rate from birth to weaning (RGR1) = ((ln (weaning weight)–ln (birth weight)) / 90 days) x 100, from weaning to six months (RGR2) = ((ln (six-month weight)–ln (weaning weight)) / 90 days) x 100 and from six months to yearling age (RGR3) = ((ln (yearling weight)–ln (six-month weight)) / 180 days) x 100.

### Statistical analysis

The general linear model procedure of SAS [16] was used to identify the important fixed effects, which have a significant effect on the efficiency-related traits. Post-hoc test was conducted using the Tukey-Kramer test. The statistical model was as follow:

$$Y_{ijklmnop}=\mu +BT_{i}+SB_{j}+SX_{k}+DP_{l}+TB_{m}+LK_{n}+GK_{o}+e_{ijklmnop}$$

Where *Y*_*ijklmnop*_ is the response variables, *μ* is the overall mean, *BT*_*i*_ is the effect of *i*^*th*^ birth type (single and multiple births), *SB*_*j*_ is the effect of *j*^*th*^ season of birth (main rain, short rain and dry), *SX*_*k*_ is the effect of *k*^*th*^ sex of kid (male and female), *DP*_*l*_ is the effect of *l*^*th*^ parity of doe (1, 2, 3, 4, and ≥5), *TB*_*m*_ is the effect of *m*^*th*^ year of birth (2009–2018), *LK*_*n*_ is the effect of *n*^*th*^ Boer blood level (25, 50, 62.5, and 75%), *GK*_*o*_ is the effect of *o*^*th*^ filial generation (F1, F2, and F3) and e_ijklmnop_ is random error term associated with each observation.

The genetic parameters were estimated by the Average Information Restricted Maximum Likelihood (AI-REML) method using WOMBAT program fitting animal model [17]. The significance of random effects was tested using the log-likelihood ratio test [18], and only the selected models are presented. An effect was considered to have a significant influence when its inclusion caused a significant increase in log-likelihood, compared with a model in which it was ignored. Phenotypic and genetic correlations among investigated traits were estimated in a bivariate analysis using starting values from univariate analyses. Genetic trends were estimated by regression of the average breeding values estimated in the particular trait on the birth year. The following six-univariate animal models were fitted:

$$y=Xb+Z_{1}a+\varepsilon$$ (1)

$$y=Xb+Z_{1}a+Z_{2}m+\varepsilon \;Cov(a,m)=0$$ (2)

$$y=Xb+Z_{1}a+Z_{2}m+\varepsilon \;Cov(a,m)=A\sigma_{am}$$ (3)

$$y=Xb+Z_{1}a+Z_{2}c+\varepsilon$$ (4)

$$y=Xb+Z_{1}a+Z_{2}m+Z_{3}c+\varepsilon \;Cov(a,m)=0$$ (5)

$$y=Xb+Z_{1}a+Z_{2}m+Z_{3}c+\varepsilon \;Cov(a,m)=A\sigma_{am}$$ (6)

Where **y** is the vector of the records of investigated traits; **b, a, m, c,** and **ε** are vectors of fixed effects, additive direct genetic, maternal additive genetic, permanent environmental effects of the dam and residual effects, respectively; **X, Z**_**1**_**, Z**_**2,**_ and **Z**_**3**_ are incidence matrices that relate these effects to the records. It was assumed that **a, m, c,** and **ε** are normally distributed with the mean zero and variance **Aσ**^**2**^_**a**_**, Aσ**^**2**^_**m**_**, I**_**p**_**c**^**2**^, and **I**_**n**_**ε**^**2**^, respectively. Where **A** is the numerator relationship matrix between animals; **σ**_**am**_ is the covariance between additive direct and maternal genetic effects; **I**_p_ and **I**_n_ are identity matrices with orders equal to the number of does and kids, respectively. **σ**^**2**^_**a,**_ **σ**^**2**^_**m,**_ **c**^**2**^, and **ε**^**2**^ are the direct additive genetic variance, maternal additive genetic variance, maternal permanent environmental variance, and residual variance, respectively.

The heritability of direct genetic effects (**h**^**2**^_**a**_) = σ^2^_a_ / σ^2^_p_; heritability of maternal genetic effects (**h**^**2**^**m**) = σ^2^_m_ / σ^2^_p_; direct-maternal genetic correlation (**r**_**am**_) was computed as the ratio of the σ_am_ to the product of the square roots of estimates of σ^2^_a_ and σ^2^_m_; maternal permanent environmental variance as a proportion of phenotypic variance (**c**^**2**^) = σ^2^_c_ / σ^2^_p_; residual variance as a proportion of phenotypic variance (**e**^**2**^) = σ^2^_e_ / σ^2^_p_. Where σ^2^_a_ is the direct genetic variance, σ^2^m is the maternal genetic variance, σ_am_ is the direct-maternal genetic covariance and σ^2^_c_ is the maternal permanent environmental variance, σ^2^_e_ is the residual variance and σ^2^_p_ is the phenotypic variance. Total heritability (**h**^**2**^_**t**_) was estimated according to Willham [19] as follows:

$${h^{2}}_{t}=({\sigma^{2}}_{a}+0.5\;{\sigma^{2}}_{m}+1.5\;\sigma_{am})/{\sigma^{2}}_{p}$$

Where σ^2^_a_ is the additive genetic variance, σ^2^_m_ is the maternal genetic variance, σ^2^_p_ is the phenotypic variance, and σ_am_ is the covariance between additive direct and maternal genetic effects.

The additive coefficient of variation (CV_A_) was computed as follows:

$${\mathrm{CV}}_{A}=\left(\sqrt{\frac{\sigma^{2}a}{ñ}}\right)x\;100$$

Where σ^2^_a_ is the additive genetic variance and ñ is the sample mean.

## Results

### Non-genetic and genetic effects

The efficiency-related traits of Boer x Central Highland goats are presented in Table 2. The least-squares means and standard errors for GE1, GE2, GE3, RGR1, RGR2, and RGR3 were 294.0 ± 5.06, 36.6 ± 1.20, 44.9 ± 1.81, 1.46 ± 0.01, 0.32 ± 0.01, and 0.19 ± 0.01, respectively. Singletons’ pre-weaning growth efficiency and relative growth rate were higher than those of multiple-born kids. However, the post-weaning efficiency for multiple-born kids was greater than for singletons. The sex of kids considerably influenced GE1, GE3, and RGR3, and male kids had a higher value for these traits than females.

**Table 2:** Efficiency-related traits (LSM ± SE) of Boer x Central Highland goats.

SV	N	GE1	RGR1	N	GE2		RGR2	N	GE3		RGR3
Overall	672	294.0± 5.06	1.46±0.01	532		36.6±1.20	0.32±0.01	350		44.9±1.81	0.19±0.01
**BT**		***	***			***	***			***	***
Single	217	317.1±8.88	1.53±0.02	174		25.7±1.68	0.24±0.01	114		42.1±3.25	0.17±0.01
Multiple	455	283.0±6.09	1.43±0.02	358		41.8±1.52	0.37±0.01	236		49.2±2.17	0.20±0.01
**BL**		*	*			*	*			ns	ns
25%	40	393.4±20.8^a^	1.73±0.05^a^	34		44.5±4.84^a^	0.39±0.03^a^	17		47.2±9.17	0.19±0.03
50%	507	292.8±5.67^b^	1.46±0.02^b^	414		36.5±1.37^ab^	0.32±0.01^ab^	278		49.1±2.08	0.20±0.01
62.50%	31	299.6±30.5^b^	1.44±0.08^b^	24		40.0±5.29^ab^	0.35±0.04^ab^	14		53.6±7.32	0.22±0.02
75%	94	256.4±11.8^b^	1.36±0.03^b^	60		31.5±3.37^b^	0.28±0.03^b^	41		29.7±3.42	0.13±0.01
**FG**		ns	ns			ns	ns			ns	ns
F1	348	284.6±6.92	1.44±0.02	298		41.2±1.64	0.36±0.01	193		50.9±2.58	0.21±0.01
F2	222	289.0±8.29	1.45±0.02	150		29.5±2.03	0.27±0.02	102		44.6±3.03	0.18±0.01
F3	102	337.2±14.5	1.57±0.04	84		33.0±3.03	0.29±0.02	55		37.1±4.04	0.16±0.01
**Parity**		**	*			ns	ns			ns	ns
1	198	296.7±9.45^ab^	1.47±0.02^ab^	149		39.4±2.22	0.35±0.01	92		57.6±3.90	0.23±0.01
2	199	313.2±9.82^a^	1.51±0.03^a^	169		36.7±2.37	0.32±0.02	115		44.2±3.25	0.18±0.01
3	146	268.2±9.72^b^	1.40±0.02^b^	113		34.7±2.54	0.31±0.02	67		36.0±3.20	0.15±0.01
4	72	280.6±14.2^ab^	1.43±0.04^ab^	59		32.5±3.04	0.29±0.02	43		52.5±4.73	0.22±0.02
≥5	57	300.5±18.1^ab^	1.48±0.05^ab^	42		36.8±3.94	0.33±0.03	33		41.2±5.06	0.18±0.02
**Season**		**	**			ns	ns			ns	ns
Dry	337	295.5±7.14^b^	1.47±0.02^b^	257		32.8±1.58	0.29±0.01	182		40.3±1.94	0.17±0.01
SR	78	335.5±16.5^a^	1.57±0.04^a^	68		45.4±4.51	0.38±0.03	32		57.6±6.54	0.23±0.02
MR	257	279.6±7.74^b^	1.42±0.02^b^	207		38.4±180	0.34±0.01	136		53.2±3.43	0.21±0.01
**Sex**		*	ns			ns	ns			**	*
Female	368	283.9±6.26	1.44±0.02	301		38.0±1.55	0.34±0.01	218		44.9±2.17	0.19±0.01
Male	304	306.2±8.18	1.49±0.02	231		34.8±1.89	0.31±0.01	132		50.1±3.18	0.20±0.01
**Year**		***	***			***	***			***	***
2009	85	263.6±13.7^cb^	1.37±0.04^bc^	80		55.3±3.54^a^	0.47±0.02^a^	51		79.3±5.69^a^	0.30±0.01^a^
2010	105	274.3±10.6^cb^	1.42±0.03^bc^	95		33.1±2.67^bc^	0.29±0.02^bc^	59		29.5±3.01^de^	0.13±0.01^de^
2011	65	216.6±9.80^d^	1.25±0.04^d^	40		27.3±2.22^cd^	0.26±0.02^cd^	32		42.0±4.17^cd^	0.18±0.01^bc^
2012	63	300.5±17.9^b^	1.47±0.04^b^	47		20.9±2.35^d^	0.20±0.02^d^	28		35.8±3.53^cd^	0.16±0.01^bcd^
2013	91	246.8±9.13^cd^	1.35±0.03^cd^	62		37.0±3.54^bc^	0.33±0.03^bc^	51		31.0±3.42^de^	0.14±0.01^cde^
2014	45	281.8±16.5^bc^	1.44±0.05^bc^	24		35.3±6.68^bc^	0.30±0.05^bc^	15		48.7±6.74^c^	0.21±0.02^b^
2016	114	376.5±12.6^a^	1.68±0.03^a^	102		40.4±2.79^b^	0.35±0.02^a^	56		64.2±4.75^b^	0.26±0.01^a^
2017	68	305.1±19.2^b^	1.47±0.05^b^	46		39.5±3.62^b^	0.35±0.03^b^	29		62.1±4.25^b^	0.25±0.01^a^
2018	36	404.8±18.0^a^	1.77±0.04^a^	36		20.7±3.09^d^	0.20±0.02^d^	29		19.6±2.01^e^	0.09±0.009^e^

SV, sources of variation; BT, birth type; BL, Boer blood level; FG, filial generation; SR, short rain; MR, main rain; GE1, growth efficiency from birth to weaning; GE2, growth efficiency from weaning to six months; GE3, growth efficiency from six- months to yearling age; RGR1, relative growth rate from birth to weaning; RGR2, relative growth rate from weaning to six months; RGR3, relative growth rate from six- month to yearling age

Ns

P>0.05; ***

P<0.001; **

P<0.01; *

P<0.05; N, number of observations

Means with different superscripts in each subclass within a column differ significantly (P < 0.05) from each other

Kids with a 25% Boer level had higher growth efficiency and relative growth rate. Likewise, they had better growth efficiency during weaning to six-month age than kids with a 75% Boer level (Table 2). The growth efficiency and relative growth rate for F1, F2, and F3 kids at all growth phases were found to be statistically similar (P>0.05). The pre-weaning growth efficiency and relative growth rate of kids born from the 2^nd^ parity were higher than 3^rd^ parity. For post-weaning efficiency-related traits, however, parity did not have a sizable effect (P>0.05). Season of kidding exerted a significant influence on pre-weaning efficiency-related traits (Table 2). Better growth efficiency and relative growth rate observed for kids born during the short rainy season than in other seasons. The year of kidding also significantly influenced all considered efficiency-related traits of the goats.

### (Co) variance components and heritability

Variance, covariance, and heritability estimates for efficiency traits in Boer x Central Highland goats are shown in Table 3. As per the log-likelihood ratio test, the selected models for GE1, GE2, GE3, RGR1, RGR2, and RGR3 were model 2, 6 2, 6, 6, and 1, respectively. About 18%, 3%, 23%, 20%, and 12% of the phenotypic variation in GE2, GE3, RGR1, RGR2, and RGR3, respectively were explained by the direct additive genetic effect. However, the contributions of kids’ own genes for variation of GE1 were low. The standard error for estimates in this study seems to be high. Except for RGR3, all investigated efficiency-related traits were under the influence of maternal genetic effect, and maternal heritability (h^2^_m_) ranged from 0.09 to 0.17. Besides, the inclusion of maternal permanent environmental effects had a significant effect on heritability estimates of GE2, RGR1, and RGR2. The direct-maternal additive genetic correlation (r_am_) for GE2, RGR1, and RGR2 were -0.93, -0.83, and -0.86, respectively. In this study, all investigated traits except for GE1 had moderate to high additive genetic coefficient of variation (CV_A_ = 10.5–29.6%).

**Table 3:** Estimates of (co) variance components and heritability for efficiency-related traits of Boer x Central Highland goats.

Trait	M	σ^2^_a_	σ^2^_m_	σ_am_	σ^2^_c_	σ^2^_e_	σ^2^_p_	h^2^_a_ ± SE	h^2^_m_ ± SE	r _am_	c^2^ ± SE	h^2^_t_	CV_A_%
GE1	2	0.29	1453			11923	13377	0.00002±0.07	0.11±0.04			0.054	0.18
GE2	6	118.1	61.4	-79.4	136	403.4	639.4	0.18±0.16	0.10±0.14	-0.93	0.21±0.10	0.046	29.6
GE3	2	24.6	97.9			656.9	779.5	0.03±0.10	0.12±0.07			0.094	10.5
RGR1	6	0.027	0.02	-0.02	0.01	0.076	0.114	0.23±0.18	0.17±0.14	-0.83	0.08±0.07	0.075	11.2
RGR2	6	0.008	0.004	-0.01	0.01	0.025	0.04	0.20±0.15	0.09±0.14	-0.86	0.20±0.10	0.063	27.9
RGR3	1	0.001				0.008	0.009	0.12±0.10				0.133	16.6

M, model; GE1, growth efficiency from birth to weaning; GE2, growth efficiency from weaning to six months; GE3, growth efficiency from six- month to yearling age; RGR1, relative growth rate from birth to weaning; RGR2, relative growth rate from weaning to six months; RGR3, relative growth rate from six- month to yearling age

σ^2^_a_, direct genetic variance; σ^2^_m_, maternal genetic variance; σ_am_, direct-maternal genetic covariance; σ^2^_c_, maternal permanent environmental variance; σ^2^_e_, residual variance; σ^2^_p_, phenotypic variance; h^2^_a_, direct heritability; h^2^m, maternal heritability; r_am_, direct-maternal genetic correlation; c^2^, maternal permanent environmental variance as a proportion of phenotypic variance; h^2^_t_, total heritability; CV_A_, additive genetic coefficient of variation

### Genetic and phenotypic correlation estimates

The phenotypic and genetic correlations among investigated efficiency-related traits are presented in Table 4. The phenotypic correlations (r_P_) ranged from -0.43 to 0.97, and the genetic correlations (r_G_) varied between -0.64 and 0.97. Growth efficiency was genetically correlated (r_G_ = 0.71 to 0.97) with the relative growth rate of goats in the same growth phase. However, the genetic correlations among pre-weaning and post-weaning efficiency-related traits were found to be antagonistic. The phenotypic correlations showed the same trend as the genetic correlations.

**Table 4:** Genetic correlation (below diagonal) and phenotypic correlation (above diagonal).

	GE1	GE2	GE3	RGR1	RGR2	RGR3
GE1		0.41±0.04	-0.23±0.06	0.97±0.01	-0.40±0.04	-0.22±0.06
GE2	-0.64±0.24		-0.20±0.05	-0.43±0.04	0.97±0.04	-0.20±0.52
GE3	-0.03±0.41	-0.24±0.53		-0.23±0.06	-0.20±0.50	0.84±0.04
RGR1	0.97±0.02	-0.60±0.20	0.21±0.61		-0.41±0.04	-0.22±0.06
RGR2	-0.60±0.26	0.71±0.36	0.02±0.62	-0.58±0.22		-0.21±0.05
RGR3	-0.04±0.52	-0.31±0.53	0.77±0.22	0.17±0.61	-0.16±0.54	

GE1, growth efficiency from birth to weaning; GE2, growth efficiency from weaning to six months; GE3, growth efficiency from six- months to yearling age; RGR1, relative growth rate from birth to weaning; RGR2, relative growth rate from weaning to six months; RGR3, relative growth rate from six- month to yearling age

### Genetic trend for efficiency-related traits

**Fig 1:**
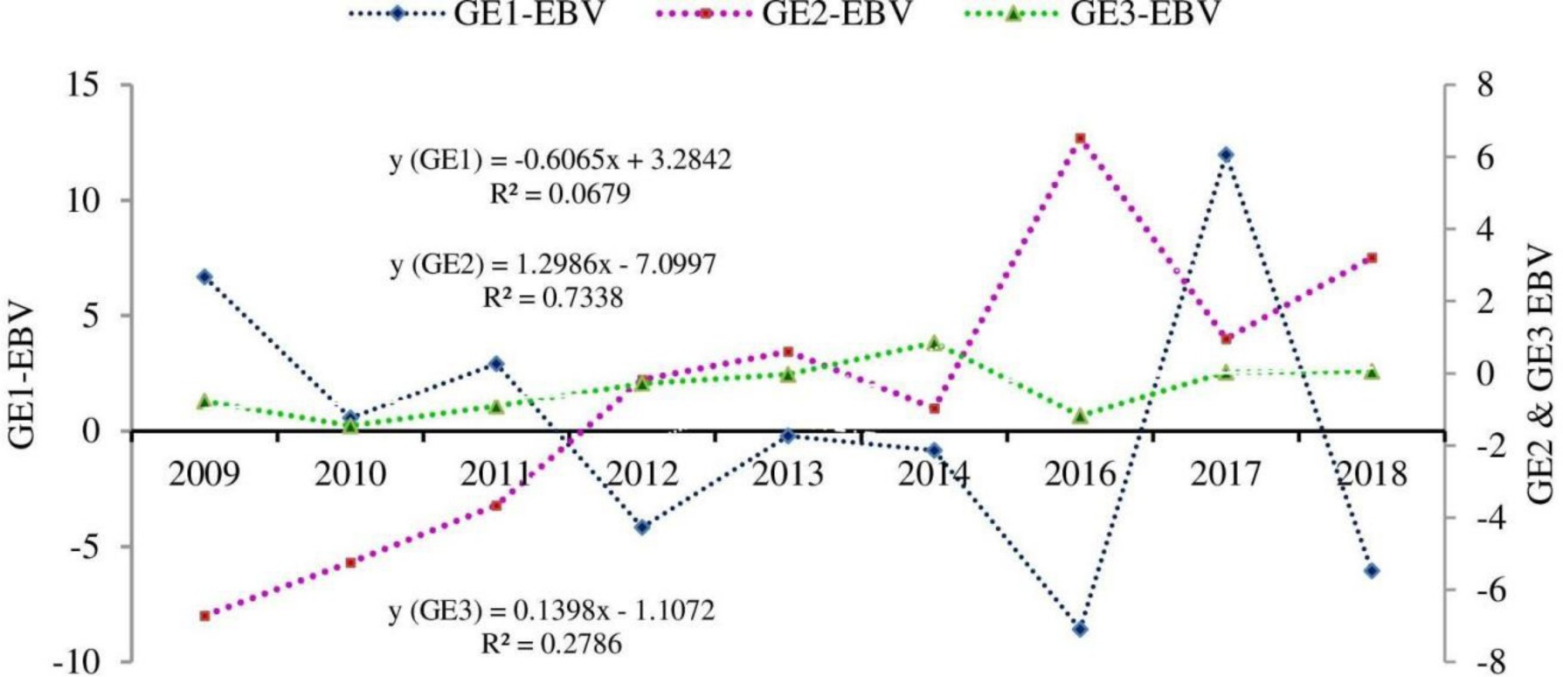
Genetic trends for growth efficiency traits at a specific age.

**Fig 2:**
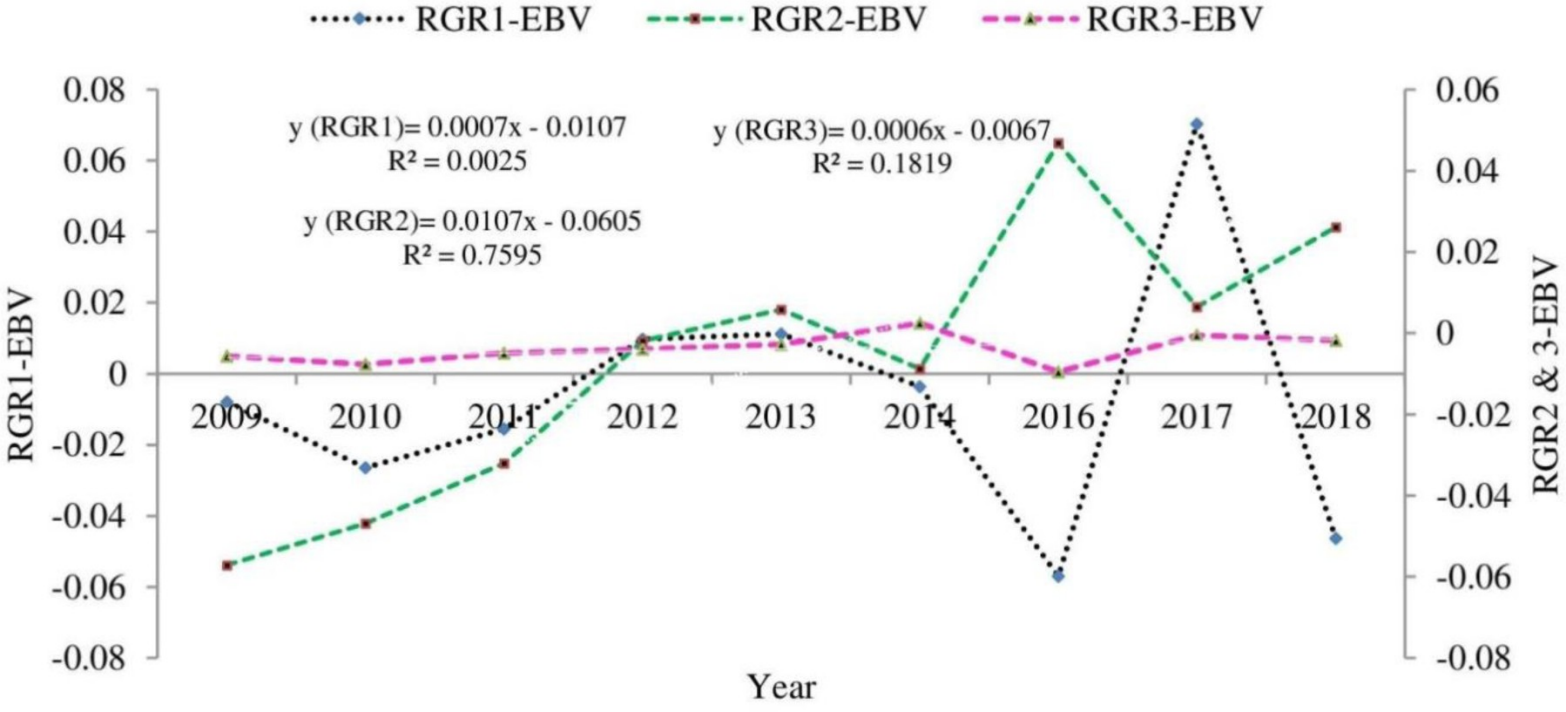
Genetic trend for relative growth rate at a specific age.

The genetic trend for growth efficiency and relative growth rate of Boer x Central Highland goats is illustrated in Figs 1 and 2, respectively. Except for GE1, the post-weaning growth efficiency showed positive genetic progress. The estimate of genetic changes for GE1, GE2, and GE3 were -0.6065, 1.2986, and 0.1398% year^-1^, respectively. The relative growth rate at different growth phases had favorable genetic progress across the years. The annual genetic changes for RGR1, RGR2, and RGR3 were 0.0007, 0.0107, and 0.1819%, respectively. In general, the estimated breeding value for investigated efficiency-related traits showed an undulating trend across the years.

## Discussion

### Systematic factors

Genetic, systematic, and non-systematic factors determine the performance of an animal. Thus, knowledge of the influence of systematic factors on efficiency-related traits is quite important for designing management and genetic improvement programs. The GE1 and GE2 in this study are lower than the report of Mokhtari et al. [13] for the Raeini Cashmere goat. However, Ghafouri-Kesbi and Gholizadeh [12] noted a relatively lower GE2, RGR2, and RGR3 in sheep than in the current study. This variability might be due to the variation in the management level and genetic potential of the breed/species. The effect of birth type on efficiency-related traits in this study is consistent with other studies [11, 12], which noted that the GE and RGR of triplet lambs were superior to single and twin lambs during post-weaning age. Besides, Mohammadi et al. [20] reported that single-born kids grew faster than multiple-born kids during the pre-weaning while they did not during the post-weaning period. However, pre-weaning and post-weaning growth efficiency for triple-born kids were higher than for single and twins, according to Mokhtari et al. [13]. Multiple-born kids are computing for resources during the pre-natal and post-natal periods, and thus, they try to adapt/survive forcefully. This forceful adaptive mechanism of kids could be the reason for their superiority during the post-weaning period.

Al-Saef [21], Ghafouri-Kesbi and Rafie-Tari [11], and Ghafouri-Kesbi and Gholizadeh [12] have reported the superiority of males over females in terms of efficiency-related traits. According to Baneh and Hafezian [22], the estrogen hormone limits the growth of long bones in females more than in males. This might be a possible reason for the inferiority of females over male kids. A filial generation did not have a significant influence on investigated traits. Boujenane et al. [23] have made a similar observation regarding the growth traits of sheep. The influence of dam parity/age was noted in previous studies [24, 25], which could be explained by dams’ degree of maturity and milk production potential. A sizable effect of season and year on efficiency-related traits was noted elsewhere [1, 13, 20, 21]. The variability of climatic variables, forage availability, and disease infestation could be the reason for observed variation across years and seasons.

### Genetic parameters

The contribution of direct additive genetic effect to phenotypic variation of pre-weaning growth efficiency (GE1) seems low relative to GE2 and GE3. This low contribution of kid’s own genes for phenotypic variation of GE1 indicates that maternal genes and genes that have a non-additive effect could control GE1. In addition, the contribution of kids’ genes is pronounced after weaning, which could be the reason for a relatively higher direct additive genetic effect after weaning age. Yogesh et al. [26] and Latifi et al. [27] have made a similar observation. The observed high standard error for estimates in this study could be ascribed to the sample size, data structure, or a small number of progeny per dam and limited information on linked offspring and dams’ performances. There are few reports of heritability for growth efficiency and relative growth rate of small ruminants in the literature. Mokhtari et al. [13] noted a relatively higher h^2^_a_ estimate for GE1 and GE3 in goats and relatively high estimate was reported by Ghafouri-Kesbi and Gholizadeh [12] in sheep than in the current study. However, the h^2^_a_ estimates for RGR1, RGR2, and RGR3 in this study are higher than the estimates reported by Ghafouri-Kesbi and Gholizadeh [12] and Ghafouri Kesbi and Rafie-Tari [11].

The maternal heritability (h^2^_m_) of GE1 in this study is well consistent with the report (0.10) of Mokhtari et al. [13]. However, Ghafouri-Kesbi and Gholizadeh (12] noted a lower estimate (0.04) for GE1 than the current result in Baluchi sheep. The estimate of h^2^_m_ for RGR1 is higher than the estimate reported by Ghafouri-Kesbi and Gholizadeh [12]. The higher h^2^_m_ estimate for GE1 and GE3 than h^2^_a_ indicates that genes controlling maternal performance (uterine nourishment, milk production, cytoplasmic effect, and mothering ability) had a higher influence on these traits than the genes carried by the kids. Likewise, genes contributing to maternal performance had a significant contribution to RGR1 and RGR2. The influence of the maternal genetic effect could be attributed to differences in the quality and capacity of the uterine space for the growth of the fetus [28]. Thus, a lack of consideration of maternal genetic effects makes it difficult to select superior goats, as the true genetic potential of kids is masked by maternal performance. Therefore, maternal effects should be included in the model to obtain accurate estimates of genetic parameters, to have accurate estimates of breeding values, and to be useful for setting selection strategies for genetic improvement.

Higher and negative additive-maternal correlations (r_am_) were noted for growth traits and Kleiber ratio by different scholars [13, 27, 29–31]. The negative r_am_ estimate may be due to natural selection for an intermediate optimum [32], poor data structure, true genetic antagonism [18], and negative environment correlation between the dam and offspring [33]. If it is due to true genetic antagonism, the negative estimate implies that selection for increasing these traits in kids unfavorably affects the maternal ability of dam for these traits. Thus, the selection program should consider both direct additive and maternal genetic effects to maximize genetic gain. In addition, Gutierrez et al. [34] suggested that the antagonistic relationship could be compensated by improving management practices and using supplementary feeding.

About 60 to 80% of the cost of animal production is feed cost [35]. Moreover, nowadays, the land use pattern has changed, i.e., grazing land is shrinking at the cost of extensive crop production, and the goat grazing/browsing resources are disappearing at a fast rate [36]. In this situation, selecting efficient animals would be imperative to ensure the sustainability of goat production. In small ruminants, an increased growth rate up to market weight with relatively little increase in mature size could help to improve production efficiency by reducing production costs. This can be achieved by selection based on relative growth rate [3, 11] and growth efficiency. However, the heritability estimates for relative growth rate and growth efficiency in this study suggest that genetic progress through selection would be slow under prevailing management conditions.

When there is a lot of residual error variance in a trait, heritability is not a viable way to measure the amount of genetic variation in the variance of a trait [5]. In such a situation, Houle [37] suggested using additive coefficients of variation, which is a measure of additive genetic variation that standardized by the trait mean and thus independent of other sources of variance, unlike heritability [38]. The moderate genetic coefficient of variation for most of the investigated traits in this study shows the possibility of including efficiency-related traits in the selection index of the breeding program to improve the efficiency of goat production. Besides heritability and genetic variability, the economic importance of a trait and its genetic correlation with other traits are important to allow a trait to be included in the selection index [39]. According to Cassel [40], improvement through mass selection (i.e., selection based on own performance) may be difficult if the heritability estimate is <0.15. In this situation, selection of goats using single source of information may not be sufficient and thus, selection using breeding values estimated from different sources of information would be important.

The genetic correlations of GE1-RGR1, GE2-RGR2, and GE3-RGR3 in this study are higher than the estimate (r_G_ = 0.429 to 0.654) reported by Ghafouri-Kesbi and Gholizadeh [12]. This means selection for higher growth efficiency would positively influence the relative growth rate of goats. The antagonistic genetic correlations among pre-weaning and post-weaning efficiency-related traits are in agreement with several studies [5, 11–13]. Therefore, selection for GE1 and RGR1 could not positively impact post-weaning efficiency traits, i.e., selection for more efficient animals in the pre-weaning age would tend to decrease the growth efficiency in the post-weaning period. This could be explained by the compensatory growth of kids. This result suggests that not all efficiency-related traits can be genetically improved concurrently through selection, and thus selection should focus on the trait of the highest importance. Nearly six months of age was when farmers sold crossbred goats. In such a scenario, improving the growth rate till the marketing age could increase Boer crossbred goat productivity efficiency.

### Genetic trend

There are no reports of genetic trends for efficiency-related traits of goats in the literature so far. Mahala et al. [31] noted positive genetic progress for other pre-weaning efficiency-related traits such as absolute weight gain (0.5477) and Kleiber ratio (0.0299). Contrary to the current study, the negative progress for absolute weight gain (-0.0451) and Kleiber ratio (-0.0065) during the post-weaning age (six months to yearling) was reported by Mahala et al. [31]. The level of selection implemented and the culling rate of animals as per their estimated breeding value explain being favorable and unfavorable genetic progress. In this study, selection and culling were not that much strong, i.e. selection was conducted based on their physical performance, and small numbers of goats were culled due to their poor performance. Thus, these issues could explain the observed undulating genetic trend for investigated trends across years. The absence of a selection of dams based on breeding value could be the possible reason for observed unfavorable genetic progress for pre-weaning growth efficiency, as the pre-weaning performance of kids is under the sizable influence of the gene controlling maternal ability.

The genetic parameter estimates are affected by animal management, breed/population, selection pressure, data size and structure. Hence, the study is limited to one flock, providing information about that specific flock. In addition, the data structure and sample size may have some influence on how the study’s conclusions are interpreted. Nevertheless, the data was collected by researchers managing the animals under on-station, which would have lessened its influence on estimates of genetic parameters. Anyways, the small dataset and data structure in this study could increase the standard error of the estimate. Thus, estimating genetic parameters using large datasets and good data structure would be important to improve estimation accuracy.

## Conclusion

Efficiency-related traits are paramount in goat production to be effective and profitable. These traits influenced by birth type, blood level, sex, season, and year of kidding. Although the heritability is low, the moderate genetic coefficient of variation and high economic importance of efficiency-related traits suggest the possibility of including these traits in the selection index to improve the efficiency of goat production. The moderate to high genetic correlation between growth efficiency and relative growth rate in the same growth phase shows the scope of multi-trait selection based on one of these traits. The selection of goats based on their breeding value would further improve genetic progress.

## Supporting information

S1 Data Goat data. (XLS)

## References

[1] Tesema Z, Tilahun M, Deribe B, Lakew M, Belayneh N, Zegeye A, et al. Effect of non-genetic factors on preweaning growth, survivability and prolificacy of Central Highland x Boer crossbred goats in North Eastern Ethiopia. Livestock Research for Rural Development. 2017; 29 (7). http://www.lrrd.org/lrrd29/7/zele29136.html.

[2] Kahia AK, Wasike CB, Bett RC. Goat breeding in low input production systems: integrating values and modern breeding technologies for improving instrinsic robustness. A paper presented at the XI International Conference on Goats in Las Palmas de Gran Canaria in the Canary Islands (Spain) September 24–27, 2012.

[3] FAO. Breeding plans for ruminant livestock in the tropics. FAO, Food and Agriculture Organization of the United Nations, Rome. 1982. http://www.fao.org/docrep/004/x6536e/x6536e04.htm.

[4] Fitzhugh HA Jr. Taylor CS. Genetic analysis of degree of maturity. Journal of Animal Science. 1971; 33:717–725. 5092769 10.2527/jas1971.334717x

[5] Ghafouri-Kesbi F, Eskandarinasab M. Heritability of relative growth rate and its relationship with growth-related traits in Afshari sheep. Genrep., (2018). doi: 10.1016/j.genrep.2018.07.006

[6] Arthur PF, Renand G, Krauss D. Genetic and phenotypic relationships among different measures of growth and feed efficiency in young Charolais bull. Livestock Production Science. 2001; 68: 131–139. 10.1016/S0301-6226(00)00243-8.

[7] Renand G, Krauss D. Genetic relationship between fattening and slaughter traits in pure bred Charolais young bulls. Proc. 7th Wld. Congr. Genet. Appl. Livest. Prod. 2002; 31: 225–228.

[8] Nkrumah JD, Basarab JA, Wang Z, Li C, Price MA, Okine EK, et al. Genetic and phenotypic relationships of feed intake and measures of efficiency with growth and carcass merit of beef cattle. Journal of Animal Science. 2007; 85: 2711–2720. doi: 10.2527/jas.2006-767 17526662

[9] Crowley JJ, McGee M, Kenny DA, Crews DH Jr., Evans RD, Berry DP. Phenotypic and genetic parameters for different measures of feed efficiency in different breeds of Irish performance-tested beef bulls. Journal of Animal Science. 2010; 88: 885–894. doi: 10.2527/jas.2009-1852 19966161

[10] Grion AL, Mercadante MEZ, Cyrillo JNSG, Bonilha SFM, Magnani E, Branco RH. Selection for feed efficiency traits and correlated genetic responses in feed intake and weight gain of Nellore cattle. Journal of Animal Science. 2014; 92: 955–965. doi: 10.2527/jas.2013-6682 24492579

[11] Ghafouri-Kesbi F, Rafiei-Tari A. Relative growth rate in sheep: heritability and relationship with absolute growth rate and body weight. Songklanakarin Journal of Science and Technology. 2015; 37: 21–27.

[12] Ghafouri-Kesbi F, Gholizadeh M. Genetic and phenotypic aspects of growth rate and efficiency-related traits in sheep. Small Ruminant Research. 2017; 149: 181–187. 10.1016/j.smallrumres.2017.02.006.

[13] Mokhtari MS, Razmkabir M, Ghiasi H, Mohammadi Y. Genetic Evaluation of Growth Rate and Efficiency Related Traits in Raeini Cashmere Goat. Iranian Journal of Applied Animal Science. 2019; 9(2): 275–282.

[14] Bangar TC, Magotra A, Yadav AS. Estimates of covariance components and genetic parameters for growth, average daily gain and Kleiber ratio in Harnali sheep. Tropical Animal Health and Production. 2020. doi: 10.1007/s11250-020-02248-z 32144658

[15] FASS (Federation of Animal Science Societies). Guide for the Care and Use of Agricultural Animals in Research and Teaching, third ed. FASS, Champaign, IL. 2010.

[16] SAS. SAS user’s guide version 9.1: Statistics. Cary, NC: SAS Institute Inc.2002.

[17] Meyer K. WOMBAT–A tool for mixed model analyses in quantitative genetics by REML. Journal of Zhejiang University–Science B. 2007; 8: 815–821. doi: 10.1631/jzus.2007.B0815 17973343 PMC2064953

[18] Meyer K. Estimates of genetic parameters for weaning weight of beef cattle accounting for direct-maternal environmental covariances. Livestock Production Science. 1997; 52: 187–199. 10.1016/S0301-6226(97)00144-9.

[19] Willham R. The role of maternal effects in animal breeding: III. Biometrical aspects of maternal effects in animals. Journal of Animal Science. 1972; 35: 1288–1293. 10.2527/jas1972.3561288x. 4567216

[20] Mohammadi H, Shahrebabak MM, Shahrebabak HM. Genetic parameter estimates for growth traits and prolificacy in Raeini Cashmere goats. Tropical Animal Health and Production. 2012; 44: 1213–1220. doi: 10.1007/s11250-011-0059-z 22213036

[21] Al-Saef AM. Genetic and phenotypic parameters of body weights in Saudi Aradi goat and their crosses with Syrian Damascus goat. Small Ruminant Research. 2013; 112: 35–38. 10.1016/j.smallrumres.2012.12.021.

[22] Baneh H, Hafezian H. Effects of environmental factors on growth traits in Ghezel sheep. African Journal of Biotechnology. 2009; 8: 2903–2907.

[23] Boujenane I, Chafik A, Benbihi M. Heterosis retained in different generations of inter se mating between D’man and Sardi sheep. Journal of Animal Breeding and Genetics.1999; 116: 151–159. 10.1046/j.1439-0388.1999.00137.x.

[24] Eskandarinasab MP, Ghafouri-Kesbi F, Abbasi MA. Different models for evaluation of growth traits and Kleiber ratio in an experimental flock of Iranian fat-tailed Afshari sheep. Journal of Animal Breeding and Genetics. 2010; 127: 26–33. doi: 10.1111/j.1439-0388.2008.00789.x 20074184

[25] Al-Bial A, Singh J, Singh DP, Niwas R. Environmental and genetic factors on growth traits of Black Bangal sheep in Yemen. The Bioscan. 2012; 7: 185–188.

[26] Yogesh C, Bangar Ankit Magotra AS. Yadav. Variance components and genetic parameter estimates for pre-weaning and post-weaning growth traits in Jakhrana goat. Small Ruminant Research. 2020; 193:106278. 10.1016/j.smallrumres.2020.106278.

[27] Latifi M, Naderi Y, Bohlouli M, Sadeghi S. Direct and maternal genetic components for body weight traits in Markhoz goat. Trop Anim Health Prod. 2021; 53(2): 234. doi: 10.1007/s11250-021-02614-5 . 33783653

[28] Gowane GR, Chopra A, Prakash V, Arora AL. Estimates of (co)variance components and genetic parameters for body weights and first greasy fleece weight in Malpura sheep. Livestock Science. 2010; 131: 94–101. 10.1016/j.livsci.2010.03.006. 22443946

[29] Gowane GR, Chopra A, Prakash Ved. Estimates of (co)variance components and genetic parameters for growth traits in Sirohi goat. Tropical Animal Health and Production. 2011; 43: 189–198. doi: 10.1007/s11250-010-9673-4 20711812

[30] Aboul-Naga AM, Hamed A, Shaat I, Mabrouk MMS. Genetic improvement of Egyptian Nubian goats as sub–tropical dairy prolific breed. Small Ruminant Research. 2012; 102: 125–130. 10.1016/j.smallrumres.2011.06.014.

[31] Mahala S, Saini S, Kumar A, Sharma RC, Gowane GR. Genetic trends for the growth rates and Kleiber ratio in Avikalin sheep. Small Ruminant Research (2020).2020. 10.1016/j.smallrumres.2020.106143.

[32] Prakash V, Prince LLL, Gowane GR, Arora AL. The estimation of (co)variance components and genetic parameters for growth traits and Kleiber ratios in Malpura sheep of India. Small Ruminant Research. 2012; 108 (2012): 54–58.

[33] Iwaisaki H, Tsuruta S, Misztal I, Bertrand JK. Estimation of correlation between maternal permanent environmental effects of related dams in beef cattle. Journal of Animal Science. 2005; 83: 537–542. doi: 10.2527/2005.833537x 15705749

[34] Gutierrez JP, Canon J, Goyache F. Estimation of direct and maternal genetic parameters for pre-weaning traits in the Asturiana de losValles beef cattle breed through animal and sire models. Journal of Animal Breeding and Genetics.1997; 114: 261–266. doi: 10.1111/j.1439-0388.1997.tb00511.x 21395821

[35] Montanholi YR, Odongo NE, Swanson KC, Schenkel FS., McBride BW, Miller SP. Application of infrared thermography as an indicator of heat and methane production and its use in the study of skin temperature in response to physiological events in dairy cattle (*Bos taurus*). Journal of Thermal Biology. 2008; 33: 468–475. 10.1016/j.jtherbio.2008.09.001.

[36] Gowane GR, Kumar A, Nimbkar C. Challenges and opportunities to livestock breeding programmes in India. Journal of Animal Breeding and Genetics. 2019; 00:1–10, doi: 10.1111/jbg.12391 30873687

[37] Houle D. Comparing evolvability and variability of quantitative traits. Genetics. 1992; 130: 195–204. doi: 10.1093/genetics/130.1.195 1732160 PMC1204793

[38] Garcia-Gonzalez F, Simmons LW, Tomkins JL, Kotiaho JS, Evans JP. Comparing evolvabilities: common errors surrounding the calculation and use of coefficients of additive genetic variation. Evolution. 2012; 66: 2341–2349. doi: 10.1111/j.1558-5646.2011.01565.x 22834736

[39] Ghafouri-Kesbi F. (Co)variance components and genetic parameters for growth rate and Kleiber ratio in fat-tailed Mehraban sheep. Archiv Tierzucht. 2013; 56 (2013): 564–572. 10.7482/0003-9438-56-055.

[40] Cassel B. Using Heritability for Genetic Improvement. Virginia Cooperative Extension, Publication 404–084, College of Agriculture and Life Sciences. Virginia Polytechnic Institute and State University, USA. 2009. http://pubs.ext.vt.edu/404/404-084/404-084_pdf.pdf [last accessed 18.12.2020].

[41] Tesema Z, Alemayehu K, Getachew T, Kebede D, Tilahun M, Deribe B, et al. Genetic analysis of efficiency-related traits in Boer x Central Highland goats. Proceedings of the 13^th^ annual regional conference on completed livestock research activities, May, 2020, Bahir Dar, Ethiopia.2021.

